# Cholesterol induces T cell exhaustion

**DOI:** 10.18632/aging.102305

**Published:** 2019-09-18

**Authors:** Xingzhe Ma, Qing Yi

**Affiliations:** 1 Center for Translational Research in Hematologic Malignancies, Houston Methodist Cancer Center, Houston Methodist Research Institute, Houston, TX 77030, USA

**Keywords:** cholesterol, T cell exhaustion, tumor microenvironment, ER stress, immune checkpoints

T cell exhaustion is a type of T cell dysfunctional state observed in chronic infections or tumor microenvironment [1]. It is often characterized by high expression of immune checkpoint receptors, decreased production of cytotoxic cytokines, decreased proliferation and altered transcriptional and metabolic profiles [2]. Although the application of immune checkpoint antibodies, which prevent or revitalize CD8^+^ T cell from exhaustion, has achieved unprecedented success, problems such as limited response rate, resistance and toxicity still exist [3]. This emphasizes the need for a better understanding of the regulatory mechanism of these immune checkpoint receptors and identifying new targeting strategies.

Cholesterol is a key component of cell membrane systems which could not only affect membrane fluidity but also cellular functions such as gene expression and metabolism [4]. The role of cholesterol in T cell activation remains controversial. Two studies showed that cholesterol or cholesterol sulfate inhibits TCR signaling by either binding to the TCRβ transmembrane region or disrupting TCR multimers, respectively [2]. Another study reported that an increase in the plasma membrane cholesterol level in CD8^+^ T cells led to enhanced TCR clustering and signaling [2]. Previously, we reported that cholesterol negatively regulates IL-9 producing CD8^+^ T cell differentiation and anti-tumor activity, and reducing cholesterol content in IL-9 producing CD8^+^ T cells enhances its IL-9 production and anti-tumor activity [5]. Recently, we found that cholesterol induces cytotoxic CD8^+^ T cell exhaustion [2], which shows another negative effect of cholesterol on T cell function.

By using different mouse tumor models, we showed that tumor tissues have a much higher cholesterol content compared with normal tissues, and the PD-1^high^2B4^high^ CD8^+^ T cells in tumor-infiltrating T cells have significantly higher cholesterol content than PD-1^med^2B4^med^ cells, which in turn have significantly higher cholesterol content than PD-1^low^2B4^low^ cells. Expression of immune checkpoints and CD8^+^ T cell exhaustion state are progressively and positively associated with cholesterol accumulation in the cells in tumor microenvironment. We also found that adoptively transferred CD8^+^ T cells acquire cholesterol and become exhausted upon entering the tumor microenvironment. The same phenomenon was observed in human multiple myeloma and colon tumor samples. In cancer patients, the expression of immune checkpoints and exhaustion state in tumor-infiltrating CD8^+^ T cell are progressively and positively associated with cholesterol accumulation in the cells. Especially, in colon cancer patients, the exhaustion state and cholesterol accumulation in tumor tissues are higher as compared with normal adjacent tissue. These indicate the clinical relevance of our study. In vitro, we found that exogenous cholesterol or cholesterol in tumor microenvironment induces CD8^+^ T cell exhaustion. Mechanistically, by using different bioinformatics approaches, we found that cholesterol induced disrupted-lipid metabolism and increased ER stress in CD8^+^ T cells. Specifically, we observed that the X-box binding protein 1 (XBP1), an ER stress sensor, was strongly induced by cholesterol treatment and strongly correlated with T cell immune checkpoint expression and T cell exhaustion. We further demonstrated that XBP1 directly induced T cell immune checkpoint expression by binding on PD-1 and 2B4 gene promoters and activated their transcription, thus leading to T cell exhaustion. Reducing CD8^+^ T cell cholesterol content or ER stress enhanced their anti-tumor activity. Reducing cholesterol content in tumor microenvironment also enhanced CD8^+^ T cell anti-tumor function [2].

However, questions remain to be answered; how does cholesterol induce ER stress in CD8^+^ T cells? Are there alternative ways that cholesterol induces T cell exhaustion other than ER stress? How do CD8^+^ T cells accumulate cholesterol and how dose tumor microenvironment accumulate cholesterol? Is it the result of de novo synthesis, transportation, or metabolism dysregulation? Further understanding of these mechanisms will help develop new therapeutic targets and methods for T cell- or immune checkpoint blockade-based immunotherapies in cancer.
